# Neutrophil Extracellular Traps Are Increased in Chronic Myeloid Leukemia and Are Differentially Affected by Tyrosine Kinase Inhibitors

**DOI:** 10.3390/cancers14010119

**Published:** 2021-12-27

**Authors:** Alona Telerman, Galit Granot, Chiya Leibovitch, Osnat Yarchovsky-Dolberg, Adi Shacham-Abulafia, Shirly Partouche, Moshe Yeshurun, Martin H. Ellis, Pia Raanani, Ofir Wolach

**Affiliations:** 1 Felsenstein Medical Research Center, Rabin Medical Center, Beilinson Hospital, Petah-Tikva 4941492, Israel; alonat55@gmail.com (A.T.); GalitG@clalit.org.il (G.G.); shirly.partouche@gmail.com (S.P.); 2 Sackler Faculty of Medicine, Tel Aviv University, Ramat-Aviv 39040, Israel; chiya.leibovitch@gmail.com (C.L.); osnat.JARCHOWSKY@clalit.org.il (O.Y.-D.); shacham.adi@gmail.com (A.S.-A.); moshe.yeshurun@gmail.com (M.Y.); martinel@clalit.org.il (M.H.E.); praanani@012.net.il (P.R.); 3 Davidoff Cancer Center, Rabin Medical Center, Institute of Hematology, Beilinson Hospital, Petah-Tikva 4941492, Israel; 4 Meir Medical Center, Hematology Institute and Blood Bank, Kfar Saba 4428164, Israel

**Keywords:** neutrophil extracellular traps, chronic myeloid leukemia, tyrosine kinase inhibitors

## Abstract

**Simple Summary:**

Neutrophil extracellular traps (NETs) are a recently described form of neutrophil cellular death that has been associated with a thrombotic tendency in many diseases. We studied NET formation in neutrophils derived from patients with chronic myeloid leukemia (CML) and in CML neutrophil cell lines and demonstrated that NETs are increased in CML and that certain drugs used to treat CML (tyrosine kinase inhibitors—TKIs) increase NET formation. These findings may shed light on a novel mechanism linking CML, TKIs and vascular toxicity.

**Abstract:**

Cardiovascular complications are increasingly reported with the use of certain tyrosine kinase inhibitors (TKIs) to treat chronic myeloid leukemia (CML). We studied neutrophil extracellular trap (NET) formation in CML and evaluated the effect of TKIs on NET formation. Neutrophils isolated from treatment-naïve patients with CML showed a significant increase in NET formation compared to matched controls at baseline and after stimulation with ionomycin (IO) and phorbol 12-myristate 13-acetate (PMA). Expression of citrullinated histone H3 (H3cit), peptidyl arginine deiminase 4 (PAD4) and reactive oxygen species (ROS) was significantly higher in CML samples compared to controls. Pre-treatment of neutrophils with TKIs was associated with a differential effect on NET formation, and ponatinib significantly augmented NET-associated elastase and ROS levels as compared to controls and other TKIs. *BCR-ABL1* retroviral transduced *HoxB8*-immortalized mouse hematopoietic progenitors, which differentiate into neutrophils in-vitro, demonstrated increased H3cit & myeloperoxidase (MPO) expression consistent with excess NET formation. This was inhibited by Cl-amidine, a PAD4 inhibitor, but not by the NADPH inhibitor diphenyleneiodonium (*DPI*). Ponatinib pre-exposure significantly increased H3cit expression in *HoxB8-BCR-ABL1* cells after stimulation with IO. In summary, CML is associated with increased NET formation, which is augmented by ponatinib, suggesting a possible role for NETs in promoting vascular toxicity in CML.

## 1. Introduction

Chronic myeloid leukemia (CML) is a myeloproliferative neoplasm (MPN) driven by the *BCR-ABL1* fusion gene. The introduction of tyrosine kinase inhibitors (TKIs) for the treatment of CML changed the natural history of this disease [1], but these drugs are also associated with some short- and long-term toxicities. Cardiovascular (CVS) complications are increasingly reported with the use of certain TKIs, such as ponatinib and nilotinib [1,2,3]. The reasons underlying these associations are not fully understood and are a matter of ongoing research, but several factors can be implicated, including TKI effect on the endothelium, on inflammatory pathways and on pro-thrombotic properties of the various blood cells [4,5,6].

In response to stimuli, neutrophils can expel extracellular strands of decondensed DNA complexed with histones, myeloperoxidase (MPO) and additional bioactive proteins [7]. These structures are able to ensnare and kill microbes but are also implicated in the pathogenesis of autoimmunity and thrombosis [8,9]. Several intracellular pathways were suggested to participate in the initiation and propagation of neutrophil extracellular trap (NET) formation. Among these, intact production of reactive oxygen species (ROS) via nicotinamide adenine dinucleotide phosphate (NADPH) oxidase [10] and peptidylarginine deiminase 4 (PAD4)-dependent NET formation seem to play a central role. Both pathways culminate in chromatin decondensation and NET formation. Increased NET formation has previously been shown to be associated with cancer-associated thrombosis [11], specifically in context of myeloid malignancies, such as Philadelphia-negative myeloproliferative neoplasms [12] and in CML mouse models [11].

In this study, we describe the characteristics and mechanism of NET formation in CML and evaluate the effect of TKIs on NET formation ex-vivo and in-vitro.

## 2. Materials and Methods

### 2.1. Human Blood Samples and Isolation of Neutrophils

Blood samples were obtained from patients with CML and from age- and gender-matched healthy controls. Whole blood was collected into ethylenediaminetetraacetic acid (EDTA)-coated tubes (vacutainers; Beckton-Dickinson, Franklin Lakes, NJ, US), and primary neutrophils were then isolated using discontinuous plasma-percoll gradients as previously described (Supplementary Materials file) [13]. Subjects presenting with conditions known or suspected to alter NET formation were excluded from the study (including active or recent infection, active cancer (in addition to CML), autoimmune diseases, current use of immunosuppressive or anti-inflammatory drugs and diabetes). The study was approved by the local ethics committee, and all participants signed an informed consent prior to enrollment.

### 2.2. BCR-ABL1 Transduced ER-HOXB8 Cell Line

ER-*HoxB8* conditionally immortalized murine myeloid progenitor cell line was differentiated into functional neutrophils as previously described (Supplementary Materials file) [14].

For creating a *BCR-ABL1* expressing neutrophil cell line, the *BCR-ABL1* oncogene was transduced into ER-*HoxB8* cells using MSCV-(pBabe mcs)-human p210*BCR-ABL*-IRES-GFP-pcDNA. For control purposes, ER-*HoxB8* cells were also transduced with an empty MSCV-(pBabe mcs)-IRES-GFP-pcDNA plasmid (Addgene, Watertown, MA, USA) (Supplementary Materials file).

### 2.3. Treatment with TKIs

Isolated human neutrophils and differentiated ER-*HoxB8* neutrophils were incubated in the presence of 5.3 µM imatinib (Selleckchem, Munich, Germany), 0.17 µM ponatinib (Selleckchem, Munich, Germany) and 0.22 µM dasatinib (Selleckchem, Munich, Germany) for 4 h. ER-*HoxB8* neutrophils were also incubated with 4.3 µM nilotinib. TKI concentrations used correspond to clinically-used oral doses of the agents [6].

### 2.4. NET Stimulation and Formation Assay

To stimulate NET formation, neutrophils (1 × 10^6^ cells per ml) were incubated with 100 nM phorbol 12-myristate 13-acetate (PMA, Sigma Aldrich, St. Louis, MO, USA) for 4 h in RPMI 1640 medium containing 10% fetal bovine serum (FBS) at 37 °C in a humidified atmosphere with 5% CO_2_. NET-bound neutrophil elastase was quantified using an available commercial kit (NETosis assay kit; Cayman Chemical, Ann Arbor, MI, USA), according to manufacturer’s instructions (Supplementary Materials file).

### 2.5. Immunostaining, Fluorescence Microscopy and NET Quantification

Procedures for immunostaining, fluorescence microscopy and NET quantification are described in the Supplementary Materials file. Briefly, neutrophils were stimulated with 5 µM ionomycin ((IO), Abcam, Cambridge, MA, USA) for 2–2.5 h at 37 °C in a humidified atmosphere with 5% CO_2_, washed and fixed with 4% paraformaldehyde (Electron Microscopy Sciences, Hatfield, PA, USA). The cells were immuno-stained overnight at 4 °C with antibodies of interest, followed by incubation with secondary antibody fluor staining. The neutrophils were next counterstained and mounted with Fluoroshield^TM^ (Sigma Aldrich), which contains 4′,6-Diamidino-2-phenylindole (DAPI) (Sigma Aldrich). Images were acquired on a fluorescence microscope. Morphologic quantification of NETs was performed on the basis of strict morphological criteria by two investigators as described in the Supplementary Materials file.

### 2.6. ROS Production Assays

Intracellular ROS levels were determined by 2′,7′-dichlorodihydrofluorescein-diacetate (DCFDA) cellular ROS detection assay kit (Abcam) as described in the Supplementary Materials file.

### 2.7. Statistical Analysis

Data are presented as mean ± SEM and were analyzed using the Student T-test or the two-tailed Mann–Whitney *U* test, as appropriate. Analyses were performed with GraphPad Prism software version 9 (San Diego, CA, USA). Results were considered significant at *p* < 0.05. Nominal *p* values were used.

## 3. Results

### 3.1. NETs Are Increased in Neutrophils from Patients with CML

**Figure 1:**
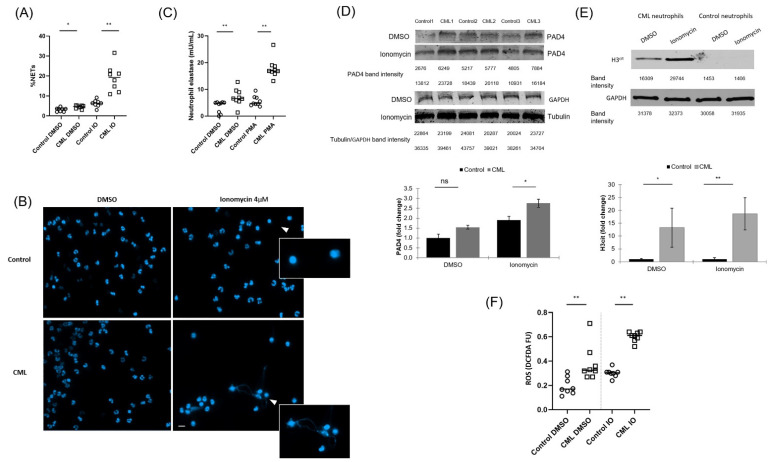
(**A**) Neutrophil extracellular trap (NET) formation in patients with newly diagnosed chronic myeloid leukemia (CML) compared to age- and gender-matched healthy controls when stimulated with 5 μM ionomycin (IO) or dimethyl sulfoxide (DMSO) for 2 h. *n* = 8 for each group. NET formation is presented as % neutrophils with morphologic changes characteristic of NET formation (see methods). * *p* < 0.05 ** *p* < 0.005. (**B**) Representative immuno-fluorescent (IF) images of neutrophils derived from patients with CML and controls after stimulation with 4 μM IO or DMSO for 2 h. 4′,6-Diamidino-2-phenylindole (DAPI) is shown in blue. White arrowheads denote NET-associated morphological changes. Scale bar, 50 μm. (**C**) NET formation expressed as the amount of NET-bound elastase in patients with newly diagnosed CML compared to healthy controls when stimulated with 100 μM phorbol 12-myristate 13-acetate (PMA) or DMSO for 4 h. *n* = 9 for each group. ** *p* < 0.005. Neutrophil lysates of patients with CML and controls blotted for (**D**) Peptidyl arginine deiminase 4 (PAD4) and (**E**) Citrullinated histone H3 (H3cit) expression after stimulation with 5µM IO or DMSO for 2.5 h. *n* = 3 for each group. * *p* < 0.05 ** *p* < 0.005. Complete blot analysis can be found in the Supplementary file (Figures S3–S5). (**F**) Reactive oxygen species (ROS) levels measured by 2′,7′-dichlorodihydrofluorescein-diacetate (DCFDA)-assay in neutrophils from newly diagnosed CML patients and from age- and gender-matched healthy controls. Neutrophils were stimulated with 5 μM IO for 2.5 h. *n* = 8 for each group. ** *p* < 0.005; FU-Fluorescence Units.

Demers and colleagues previously demonstrated that neutrophils from a retroviral transduced *BCR-ABL1* murine model have an increase in NET formation [11]. To determine whether neutrophils from CML patients are more prone to form NETs, we analyzed the ability of neutrophils isolated from patients with newly-diagnosed, treatment-naive CML to undergo NET formation and compared them to age- and gender-matched controls (*n* = 8 for each group; Table S1). NET formation was significantly increased in neutrophils derived from patients with CML at baseline and even more so after stimulation with IO as assessed by typical morphological changes (*p* < 0.05 & *p* < 0.005, respectively; Figure 1A,B), and after stimulation with PMA as assessed by quantification of NET-associated elastase (*p* < 0.005 & *p* < 0.005 before and after stimulation, respectively; Figure 1C), as compared to controls. A significant increase in NET formation was noted between dimethyl sulfoxide (DMSO) and IO treated CML neutrophils as assessed by morphology and NET-associated elastase (*p* < 0.005 for both comparisons); a significant increase in NET formation was also noted between DMSO and IO treated control neutrophils as assessed by morphology (*p* < 0.005) but not by the NET-associated elastase assay (*p* = 0.2). Increased expression of PAD4 and its downstream product, citrullinated histone 3 (H3cit) (Figure 1D,E), were also significantly increased in neutrophils derived from patients with CML. Additionally, ROS generation by NADPH oxidase, an established driver of NET formation, was significantly increased in neutrophils from patients with CML as compared to age- and gender-matched controls (*p* < 0.005 for both resting and stimulated conditions; Figure 1F).

### 3.2. Ponatinib Augments NET Formation in Neutrophils Derived from Patients with CML

**Figure 2:**
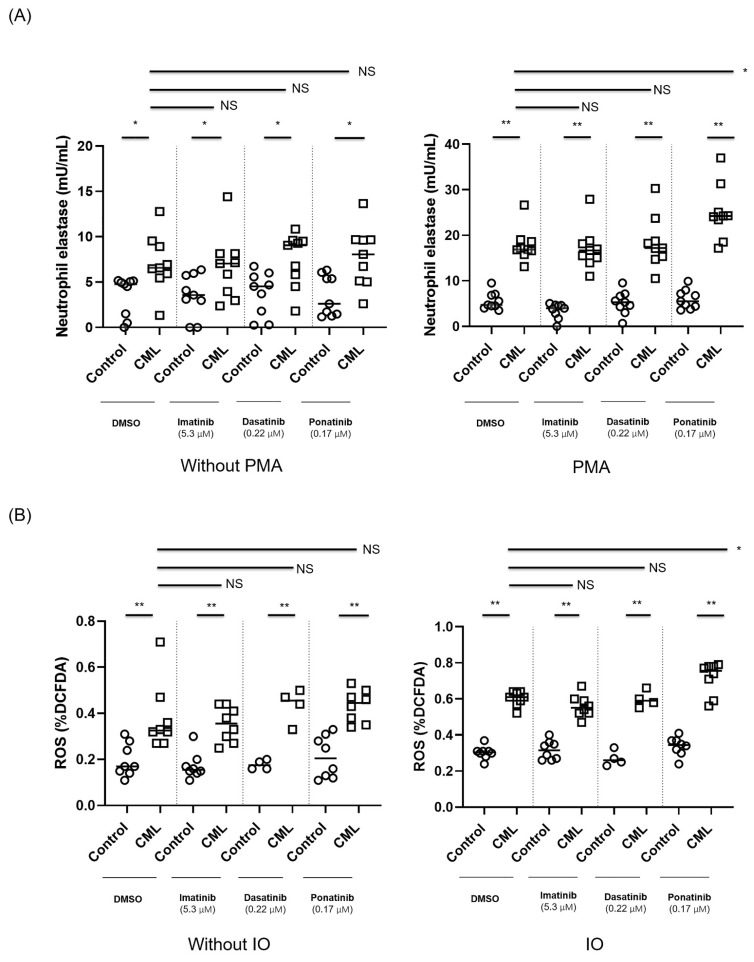
(**A**) Neutrophils from newly diagnosed CML patients and from age- and gender-matched controls (*n* = 9 for each group) were treated ex-vivo with various tyrosine kinase inhibitors (TKIs). NET formation expressed as the amount of NET-bound elastase in patients with newly diagnosed CML compared to healthy controls when stimulated with 100 μM PMA or DMSO for 4 h. * *p* < 0.05 ** *p* < 0.005; ns—non-significant. (**B**) ROS levels measured by DCFDA-assay in neutrophils from newly diagnosed CML patients and from age- and gender-matched healthy controls after exposure to various TKIs and stimulation with 4 μM IO. ROS levels were measured by flow cytometry. *n* = 8 for control, imatinib and ponatinib; *n* = 4 for dasatinib. Each dot represents the mean of one patient/control triplicate. Upper bars relate to differences between CML cells treated with DMSO (control) as compared to those treated with the different TKIs. Lower bars depict the differences between control and CML cells for each condition. The following concentrations of TKIs were used: 5.3 µM imatinib, 0.22 µM dasatinib and 0.17 µM ponatinib. * *p* < 0.05 ** *p* < 0.005; ns—non-significant.

We hypothesized that NET production may play a role in the tendency for CVS that are associated with the use of TKIs. To that end, we evaluated the effect of imatinib, dasatinib and ponatinib on NET formation in neutrophils derived from patients with CML that were stimulated with PMA or DMSO (negative control) in comparison to neutrophils from healthy subjects. Treatment of isolated, activated neutrophils with ponatinib was associated with a significant increase in NET formation as compared to DMSO-treated neutrophils as assessed by an increase in the presence of NET-bound elastase (Figure 2A; *p* ≤ 0.005).

We also demonstrate that the already elevated ROS level in neutrophils derived from patients with CML is further augmented ex-vivo by ponatinib exposure, an effect that was not demonstrated for other TKIs tested (Figure 2B). These preliminary findings suggest that exposure to ponatinib augments NET production, potentially via increased ROS production. Of note, the increase in the presence of neutrophil elastase and in ROS production seen following exposure to ponatinib are not a result of changes in the viability of the cells, as ponatinib was not shown to impact neutrophil cell viability [15].

### 3.3. BCR-ABL1 Transduced ER-HoxB8 Cell Line System Recapitulates NET Phenotype in CML, TKI Effects and Reveals PAD4 Dependency

To determine whether *BCR-ABL1* expression is responsible for promoting NET formation and to better characterize the effect of TKIs on NET formation in a stable, controlled environment, we established a *BCR-ABL1* expressing cell line with the ability to differentiate into neutrophils. We used the ER-*HoxB8* cells, which are a conditionally immortalized murine myeloid progenitor cell line, with estrogen-dependent production of *HoxB8*, a crucial transcription factor blocking myeloid differentiation. ER-*HoxB8* cells efficiently differentiate into functional neutrophils upon estrogen withdrawal and addition of stem-cell factor (SCF) [14] (Figure S1).

**Figure 3:**
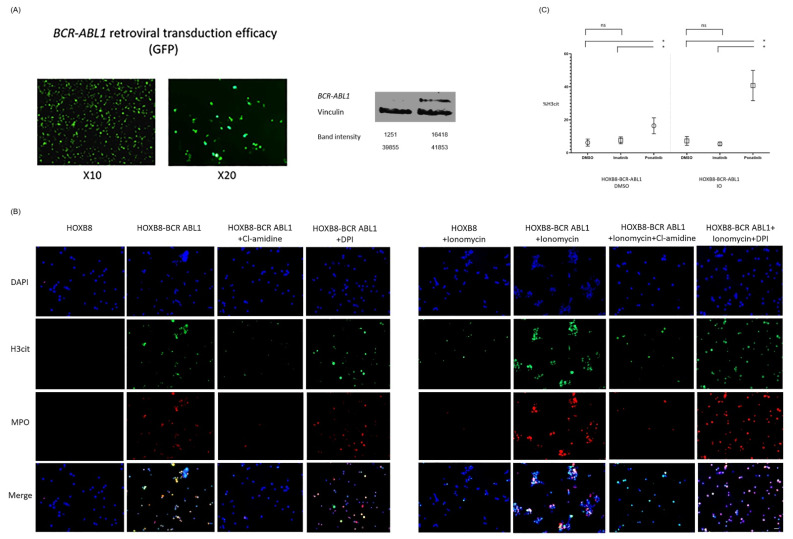
(**A**) Green fluorescent protein (GFP) and *BCR-ABL1* expression to assess retroviral transduction efficacy of *HoxB8* cells using the MSCV-(pBabe mcs)-human p210*-BCR-ABL*-IRES-GFP system. Complete blot analysis can be found in the Supplementary file (Figure S6). (**B**) *HoxB8*-*BCR-ABL1* cells and *HOXB8* cells transduced with an empty vector were stimulated with IO and treated or not with Cl-amidine, a PAD4 inhibitor (10 µM) or diphenyleneiodonium (DPI), a nicotinamide adenine dinucleotide phosphate (NADPH) oxidase inhibitor (14 µM) and stained with DAPI, anti-H3cit and anti-myeloperoxidase (MPO). Scale bar, 100 μm. (**C**) Percent of H3cit in *HoxB8-BCR-ABL1* cells and *HoxB8* cells transduced with an empty vector stimulated with 4 μM IO for 2 h and exposed to DMSO, imatinib (5.3 µM) or ponatinib (0.17 µM). * *p* < 0.05; ns—non-significant.

In order to adjust the ER-*HoxB8* system to our experimental design, we transduced the ER-*HoxB8* cells with a *BCR-ABL1* expression vector to create a *BCR-ABL1* expressing ER-*HoxB8* cell line (Figure 3A and Figure S2).

Consistent with our ex-vivo results, initial studies show that our *HoxB8*-*BCR-ABL1* cell line demonstrates an increase in H3cit and MPO expression. In addition, we showed co-localization of DNA (DAPI stained) with MPO and H3cit, a trait characteristic of NET formation. Exposing the cells to a PAD4 inhibitor, Cl-amidine, resulted in a dramatic decrease of H3cit and MPO expression. In contrast, inhibiting NADPH oxidase did not alter the expression of H3cit and MPO (Figure 3B).

Pre-treating *HoxB8-BCR-ABL1* cells and *HOXB8* cells transduced with an empty vector with different TKIs resulted in TKI-dependent differential response. In the *BCR-ABL1* transduced cells, ponatinib significantly increased H3cit expression as compared to DMSO and imatinib-treated cells in an unstimulated condition (*p* < 0.05 for all comparisons). In the stimulated condition, ponatinib also significantly increased H3cit expression as compared to DMSO and imatinib-treated cells (*p* < 0.05 for all comparisons) (Figure 3C).

Taken together, these data suggest that in the *HoxB8* cell system, *BCR-ABL1* is associated with increased NET formation that is further augmented by ponatinib in a PAD4-dependent fashion.

## 4. Discussion

The role of NETs has been studied across a wide range of pathologies, including cancer associated thrombosis [8,11], inflammatory conditions [16] and infectious states [17,18]. Philadelphia-negative myeloproliferative neoplasms have been shown to be associated with NET formation that contributes to the pro-thrombotic phenotype in murine models [12].

Demers and colleagues previously showed that neutrophils from a retroviral transduction *BCR-ABL1* murine model have an increase in NET formation [11]. We demonstrate here, to our knowledge for the first time, that neutrophils from patients with CML demonstrate an increase in NET formation at baseline and even more so after stimulation. This was associated with increased expression of PAD4 and its downstream biomarker H3cit, as well as increased production of ROS. Of note, elevated levels of ROS were previously described in CML and are both associated with *BCR-ABL1* transformation and are a marker of advanced disease and resistance to TKIs [19,20].

Cardiovascular and thrombotic adverse events may occur in patients with CML treated with TKIs, such as nilotinib and ponatinib [2], and several potential mechanisms for this association were previously proposed. Vascular endothelial dysfunction may result in upregulation of various cyto-adhesive molecules and promote a ’pro-atherogenic´ phenotype [5,6,21,22]. Latifi et al. suggested Von Willebrand Factor-mediated platelet adhesion and a secondary microvascular angiopathy induced by ponatinib exposure [23]. Additionally, several reports implicate potential off-target effects of the various TKIs on platelet, macrophage/monocytes and other myeloid cells relevant to pathogenesis of TKI-related thrombosis [2,24].

We demonstrated that exposing neutrophils from patients with CML ex-vivo to clinically relevant concentrations of TKIs resulted in a differential effect with an increase in markers of NET formation after ponatinib exposure, providing a potential novel mechanism linking ponatinib to vascular toxicity. Vascular events can occur early or late in the course of TKI therapy in CML [3]. Since the relative contribution of NET formation to vascular events may be dependent on disease burden and other factors, further studies to assess NET dynamics during TKI therapy may be revealing.

The fact that ponatinib increased NET formation is somewhat surprising since in previous studies, inhibiting kinases generally exert repressive effects on NET formation as was shown for JAK1/2 [12], CDK [25] and Raf-MEK-ERK pathway inhibition [26]. One possible explanation is our observation that in contrast to the inhibitory or neutral effects on ROS formation of JAK [27] and CDK [25] pathway inhibition, respectively, ponatinib actually increased ROS levels in neutrophils, possibly promoting NET formation, as ponatinib is a known mitochondrial toxin that impairs the function of the respiratory chain and increases ROS production [28]. Additional mechanistic insights as to the processes leading to increased NETosis succeeding ponatinib exposure in *BCR-ABL1* neutrophils are warranted.

The use of the ER-*HoxB8* cell line differentiated into neutrophils represents a novel approach to assess NET formation in a controlled in-vitro environment. Since it is technically challenging to reliably detect morphologic changes characteristic of NET formation in this cell line, we assessed H3cit expression and ROS production as surrogates for NET formation in this system. We were able to recapitulate increased NET formation and ROS production in *BCR-ABL1* transduced ER-*HoxB8* cells, validating our findings from human neutrophils. Interestingly, in this model, H3 citrullination was dependent on PAD4 expression and not on ROS production. These differences between ex-vivo human neutrophils and the murine-derived ER-*HoxB8* cells may be attributed to different degrees of dependence on ROS and PAD4 for NET formation in human and murine models. Human neutrophils are largely dependent on an intact NADPH oxidase-ROS axis [10,29] while NET formation in murine neutrophils was shown to be dependent on PAD4 expression [12,30,31]. These potential mechanistic differences should be taken into account when using murine-based models to study NET formation.

## 5. Conclusions

In summary, we report, for the first time, increased NET formation in neutrophils from patients with CML and provide evidence for differential effects of TKIs on NET formation, possibly via altered ROS production and PAD4 expression. We present a novel approach to assess NETs in-vitro using a *HoxB8* cell system and recapitulated a NET-prone phenotype in *BCR-ABL1* transduced ER-*HoxB8* cells that is PAD4-dependent.

These data further our understanding of potential processes linking ponatinib to CVS complications in CML and provide potential targets for disruption to mitigate these adverse drug effects.

## Data Availability

Data supporting reported results will be provided upon reasonable request.

## Supplementary Materials

The following are available online at www.mdpi.com/article/10.3390/cancers14010119/s1, Table S1: Clinical characteristics of CML patients assessed for NET formation. Figure S1: Differentiating *HoxB8* GMPs to neutrophils, Figure S2: Outline of *BCR-ABL1* transduced ER-*HoxB8* cell line preparation, Figures S3–S6: Original Western blots.
