# Supplementary material for: Neutrophil Extracellular Traps Are Increased in Chronic Myeloid Leukemia and Are Differentially Affected by Tyrosine Kinase Inhibitors
Source: Cancers (Basel). 2021 Dec 27;14(1):119. doi: 10.3390/cancers14010119 (PMC8750902; doi:10.3390/cancers14010119)
Supplement: Supplementary file 1 [file cancers-14-00119-s001.zip › cancers-1491840-Supplementary materials.pdf]

Table S1. Clinical characteristics of CML patients assessed for NET formation.

UPN	Age (years)	M/F	WBC at Dx (X10^9/L)	Dx	Transcript	EUTOS risk	Co-morbidities
1	53	M	178.6	CML-CP	B3A2	High	None
2	74	F	39.5	CML-CP	B3A2	Intermediate	HTN
3	27	M	30	CML-CP	B3A2	Low	None
4	57	M	140	CML-CP	B3A2	Low	Smoker
5	59	M	38	CML-CP	B3A2	Low	Fatty liver
6	25	M	67.1	CML-CP	B3A2	Low	None
7	42	F	36	CML-BP	B2A2	--	None
8	76	M	40.8	CML-CP	B3A2	Intermediate	HTN, BPH
9	70	M	14	CML-CP	B3A2	Low	IHD, HTN, Dyslipidemia, BPH, OA, Gout

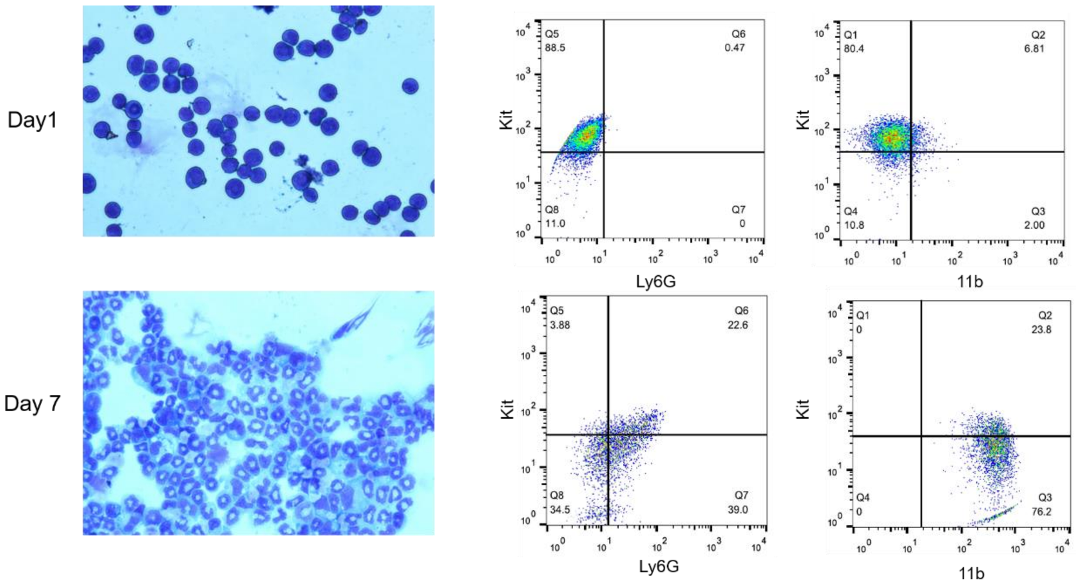

Figure S1. Upper panel demonstrates *HoxB8* cells that are positive for the stem cell marker, c-KIT. Following removal of estrogen from media and addition of SCF and GM-CSF, cells differentiate into mature murine neutrophils. Lower panel demonstrates mature 'donut-shaped' murine neutrophils that lose c-kit expression and acquire the mature neutrophil markers CD11b and Ly6G. Gimsa stain, scale X40.

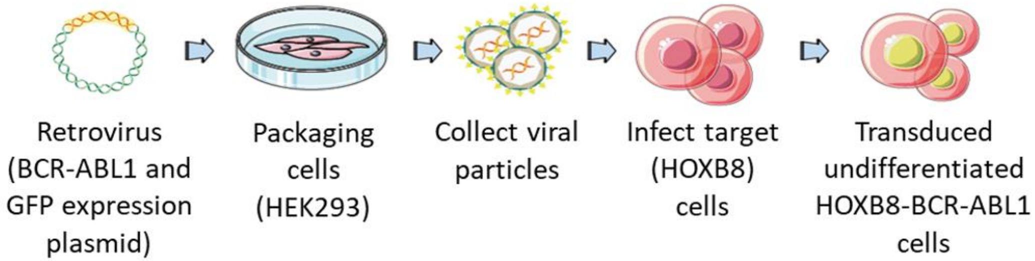

Figure S2. Outline of *BCR-ABL1* transduced ER-*HoxB8* cell line preparation.

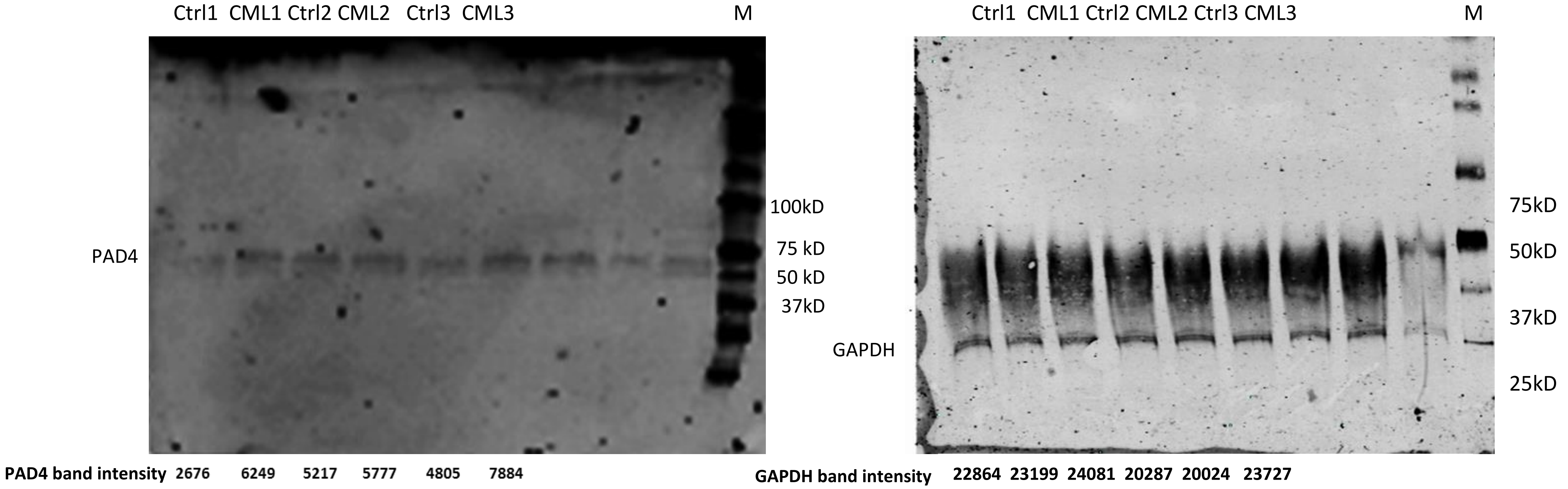

Figure S3. Western blots showing PAD4 and GAPDH expression supporting Figure 1D. Full images of the Western blot for PAD4 and tubulin expression in DMSO treated neutrophil lysates of patients with CML and controls, panels 1, 3 of Figure 1D. M - Molecular weight marker shown on the left of each blot. Ctrl – control cells.

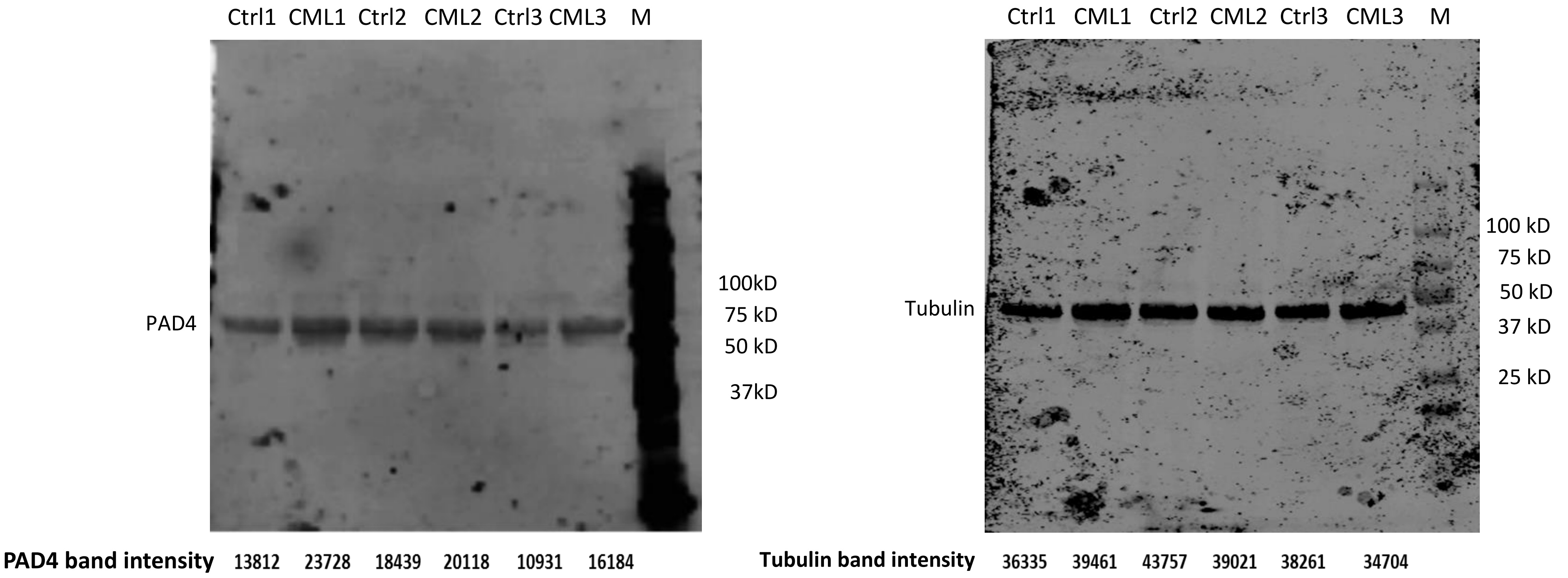

Figure S4. Western blots showing PAD4 and tubulin expression supporting Figure 1D. Full images of the Western blot for PAD4 and tubulin expression in IO treated neutrophil lysates of patients with CML and controls, panels 2, 4 of Figure 1D. M - Molecular weight marker shown on the left of each blot. Ctrl – control cells.

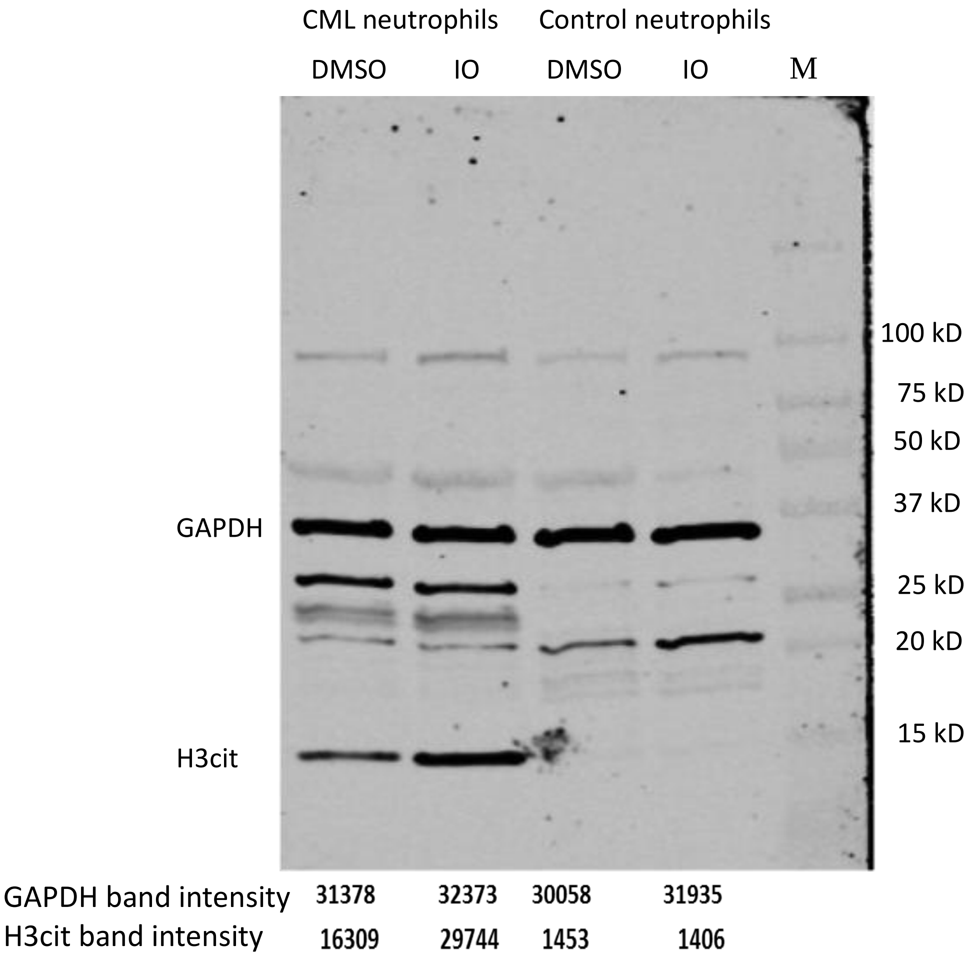

Figure S5. Western blot showing H3cit and GAPDH expression supporting Figure. 1E Full images of the Western blot for H3cit and GAPDH expression in DMSO/IO treated neutrophil lysates of patients with CML and controls shown in Figure 1E. M - Molecular weight marker shown on the left of the blot.

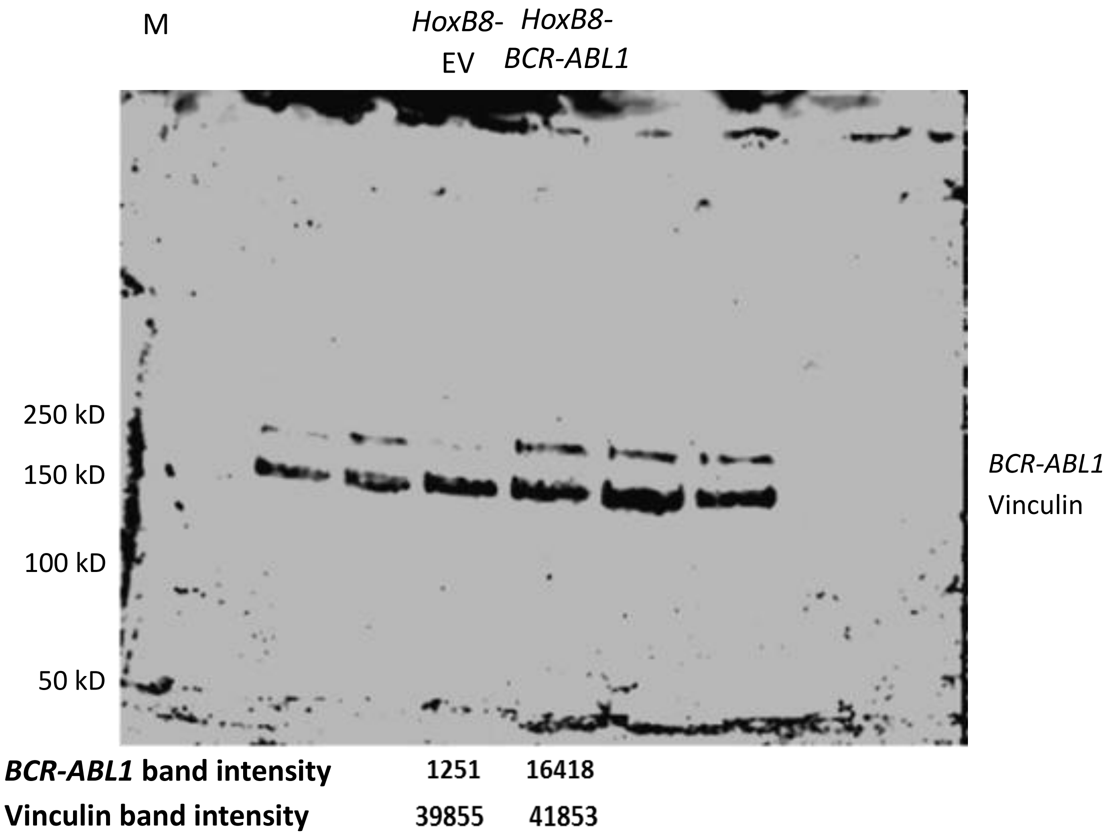

Figure S6. Western blot showing *BCR-ABL1* and vinculin expression supporting Figure 3A. Full images of the Western blot for *BCR-ABL1* and vinculin expression in *HoxB8-BCR-ABL1* cells and *HoxB8* cells transduced with an empty vector shown in Figure 3A. M - Molecular weight marker shown on the left of the blot. EV – empty vector.

Supplementary Materials:

Isolation of Human Neutrophils:

Primary neutrophils were obtained from whole blood samples using discontinuous plasma-percoll gradients as previously described (1). Briefly, 2 ml whole blood were gently loaded onto a discontinuous gradient of Percoll®PLUS (Santa Cruz Biotechnology, Dallas, Tx, USA) and centrifuged for 30 min at 1000 g. The fractions containing neutrophils, red blood cell pellet were removed and incubated for 5 min with 2 ml of RBC Lysis Buffer (Invitrogen, Dublin, Ireland) and then centrifuged at 300 g for 5 min. The supernatant was harvested and resulting pellet of neutrophils was washed once in Dulbecco's phosphate buffered saline (PBS) without calcium and magnesium (Biological Industries, Cromwell, CT, USA), then resuspended in RPMI 1640 medium (Biological Industries) supplemented with 10% fetal bovine serum (FBS) (Biological Industries) and 1% pen strep (Biological Industries). Neutrophil purity was determined by labeling with anti-human CD11b (SouthernBiotech, Birmingham, AL, USA) and measuring CD11b+ cells by Flow cytometer (Beckman Coulter, Gallios, IN, USA).

***BCR-ABL1* Transduced ER-*HoxB8* Cell Line**

The ER-*HoxB8* line was kindly provided by Dr. David B. Sykes, Massachusetts General Hospital, Harvard Medical School, Massachusetts, USA and Prof. Shai Izraeli, Hematology-Oncology Department, Schneider Children's Medical Center, Rabin Medical Center, Israel. The cells were cultured in ER-*HoxB8*-medium (RPMI 1640 medium with glutamine supplemented with 10% FBS, 1% pen strep, 10% β-estradiol (Sigma Aldrich**,** St. Louis. MO, USA) and 1% murine GM-CSF (secreted by CHO cells that were kindly provided by Dr. David B. Sykes, and Prof. Shai Izraeli), collected and stored at –80°C and seeded in 6-well culture plates at 37°C with 5% CO_2 in a humidified incubator. To differentiate ER-*HoxB8* progenitors to neutrophil-like cells, 0.5×10^6 cells/ml were washed twice with PBS, resuspended in the same ER-*HoxB8*-medium, but without β-estradiol and cultured at 37°C with 5% CO_2 in a humidified incubator. In the absence of estrogen, the cells were terminally differentiate to neutrophils after 72 hr. Prior to experiments the cells were labeled with mouse anti-neutrophil antibody PE-conjugated (Abcam, Cambridge, MA, USA) and tested by FACS for purity of neutrophil population (2).

For creating a *BCR-ABL1* expressing neutrophil cell line the *BCR-ABL1* oncogene was transduced into ER-*HoxB8* cells using MSCV-(pBabe mcs)-human p210*BCR-ABL*-IRES-GFP-pcDNA. For control purposes ER-*HoxB8* cells were also transduced with an empty MSCV-(pBabe mcs)-IRES-GFP- pcDNA plasmid (Addgene, Watertown, MA, USA).

293T HEK cells (American Type Culture Collection, ATCC, Manassas, VA, USA) were grown to 70-80% confluency in DMEM (Biological Industries) supplemented with 10% FBS and 1% pen strep. 3.5 x10^6 cells were incubated for 24 hrs with 30 µl Lipofectamine 2000 (Thermo Fisher Scientific, Waltham, MA, USA), 5 µg retroviral construct and 5 µg pCL-Eco Retrovirus Packeging Vector (NOVUS Biologicals, Centennial, CO, USA). After the incubation the medium was aspirated, filtered into 1 ml aliquots and frozen or immediately added to 25x 10^4 ER-*HoxB8* cells with 8 µg/ml polybrene (Sigma Aldrich). After 24 or 48 hrs the transduction mix was removed and the cells were centrifuged for 90 min at 1000 g at 22°C. Following the spin, fresh media was added to each well. Transduction efficiency was tested by measurement of GFP levels *via* FACS and fluorescent microscopy and was found to exceed 80%.

Stimulation of NET Generation

To stimulate NET formation, neutrophils (1 x 10^6 cells per ml) were incubated with 100 nM phorbol 12-myristate 13-acetate (PMA, Sigma Aldrich) for 4 hrs or 5 µM IO (Abcam) in RPMI 1640 medium containing 10% FBS for 2-2.5 hrs at 37 °C in a humidified atmosphere with 5% CO_2.

NET Formation Assay

For studying the process of NET formation *ex-vivo*, NET-bound neutrophil elastase was quantified using an available commercial kit (NETosis assay kit; Cayman Chemical, Ann Arbor, MI, USA), according to manufacturer’s instructions. Neutrophils were stimulated to release NETs with either PMA or IO. Unbound neutrophil elastase was washed away following NET generation. Following digestion of NET DNA by nuclease, the supernatant containing neutrophil elastase was added to a substrate, which is selectively cleaved by elastase to yield a 4-nitroaniline product. Absorbance was read at 405 nm.

Immunostaining, Fluorescence Microscopy and NET Quantification

Unstimulated and IO stimulated neutrophils (1 X 10^6) were washed twice with PBS then fixed with 4% paraformaldehyde (Electron Microscopy Sciences, Hatfield, PA, USA) for 10 min at room temperature. The cells were washed again twice and resuspended in 0.5 ml PBS. The cells were immuno-stained overnight at 4 °C with the following antibodies: rabbit anti–histone H3 (citrulline 2, 8, 17) antibody (Abcam) and rabbit anti–PAD4 antibody (Proteintech, Rosemont, IL, USA). Then cells were washed twice and incubated with Alexa 488-conjugated goat anti-rabbit IgG (Invitrogen) or Alexa 594-conjugated donkey anti-rabbit IgG (Invitrogen), both diluted 1:1000 for 1 hr at room temperature. Cells were washed again with PBS and 5 x 10^4 cells were plated on poly-L-lysine coated slides (Sigma Aldrich). The neutrophils were next counterstained and mounted with Fluoroshield™ (Sigma Aldrich) which contains 4′,6-Diamidino-2-phenylindole (DAPI) (Sigma Aldrich). Images were acquired on a fluorescence microscope Axio Imager 2 (Zeiss, Germany) or on a confocal microscope TCS SP5 (Leica, Wetzlar, Germany) using a 488 nm Argon laser (green fluorescence) or 594 nm Argon laser (red fluorescence) and a 405 nm LD laser (DAPI).

Morphologic quantification of NETs was performed on the basis of any of the following morphological criteria: nuclear delobulation, nuclear swelling or extension of web-like DNA strands. Percentages of H3cit cells and NETs were determined from three to four non-overlapping fields per well, and the average was taken from duplicates or triplicates for each condition in every experiment. NET quantification was assessed independently by two investigators (O.W. and A.T.). Results obtained by the first investigator were independently verified by a second investigator blinded to the results.

ROS Production Assays

Intracellular ROS levels were determined by 2′,7′-dichlorodihydrofluorescein-diacetate (DCFDA) cellular ROS detection assay kit (Abcam). Isolated neutrophils were pre-incubated with TKIs for 4 hrs and then loaded with DCFDA (20 µM) for 20 min. After washing extracellular DCFDA, cells were resuspended in fresh RPMI media and 1000 µL containing 350,000 cells were seeded onto 24-well plates. These cells were stimulated or not with IO for an additional duration of 180 min after which ROS production was analyzed by an ELISA Plate Reader (Epoch, BioTek, UK).

In some experiments with the transduced ER-*HoxB8* neutrophils, prior to IO stimulation, the cells were exposed to 10 µM of the NADPH inhibitor, diphenyleneiodonium chloride (DPI) (Sigma-Aldrich) for 30 min at 37°C.

Western Blotting

Neutrophils were homogenized in RIPA buffer (Sigma Aldrich) supplemented with protease inhibitor cocktail (Roche, Indianapolis, IN, USA) on ice. Protein concentration was measured by Pierce™ BCA Protein Assay kit (Thermo Fisher Scientific). Equal amounts of protein were separated on Mini-PROTEAN precast gels (Bio-Rad, Hercules, CA, USA) and transferred to a nitrocellulose Trance-Blot Turbo Transfer Pack membrane (Bio-Rad) then blotted with primary antibodies: anti–H3cit, anti–PAD4, anti–NOX4 and anti- GAPDH (Santa Cruz Biotechnology) at 4ºC overnight and subsequently with the secondary antibody: HRP-conjugated anti-rabbit IgG (Bio-Rad) and then detected with Odissey CLx (LiCore, Lincoln, NB, USA).

References:

1. Gonzalez, A.S.; Bardoel, B.W.; Harbort. C.J.; Zychlinsky, A. Induction and quantification of neutrophil extracellular traps. *Methods Mol. Biol.* **2014**,*1124*, 307–318. doi:10.1007/978-1-62703-845-4_20. Cited in: Pubmed; PMID 24504961.

2. Wang, G.G.; Calvo, K.R.; Pasillas, M.P.; Sykes, D.B.; Häcker, H.; Kamps, M.P. Quantitative production of macrophages or neutrophils ex vivo using conditional Hoxb8. *Nat. Methods* **2006**, *3*, 287–293. doi:10.1038/nmeth865. Cited in: Pubmed; PMID 16554834.
